# Factors influencing the level of patients using the internet to gather information before anaesthesia: a single-centre survey of 815 patients in Switzerland: The internet for patient information before anaesthesia

**DOI:** 10.1186/s12871-017-0319-1

**Published:** 2017-03-08

**Authors:** T. Wieser, M. P. Steurer, M. Steurer, A. Dullenkopf

**Affiliations:** 1 Department of Anaesthesiology and Intensive Care Medicine, Kantonsspital Frauenfeld, Frauenfeld, Switzerland; 2 Department of Anesthesia and Perioperative Care, UCSF, San Francisco, USA; 3 Department of Pediatrics, UCSF, San Francisco, USA

**Keywords:** Anaesthesia, Preoperative information, Internet

## Abstract

**Background:**

Aim of this study was to identify factors associated with patients using the internet to find information about their upcoming surgery in general, and more specifically about anaesthesia.

**Methods:**

With Ethics committee approval, 1000 consecutive patients seen before elective surgery in the anaesthesia preoperative clinic of a Swiss Level 2 hospital were asked to complete a questionnaire. Primary outcome were patients using the internet to gather any medical information related to their upcoming hospital stay, secondary outcome patients using the internet to gather information regarding the upcoming anaesthesia. Multiple regression was performed to identify independent factors associated with internet use.

**Results:**

Eighty-two percent of the patients (*n* = 815) participated. 97% of those were ASA physical status 1 or 2; 83% (*n* = 676) had experience with previous anaesthetics, 86% (*n* = 700) reported to use the internet in general. Overall, about one-third of the participants used the internet to learn more about their medical condition, 26% regarding their upcoming surgical procedure. Only 7% (*n* = 55) obtained information about the anaesthetic. In multivariate analyses, factors associated with internet use were generally doing so, and planned moderate compared to minor surgery; not using the internet was associated with previous anaesthetic experience. Of those who did not use the Internet to learn about their anaesthetic, 34% indicated that they would have visited a trusted website.

**Conclusion:**

Only few patients used the internet to obtain information about their upcoming procedure and the anaesthetic part played an even smaller role. However, many patients would have appreciated guidance to find trustworthy internet sites.

**Trial registration:**

German Clinical Trials Register (DRKS00005434; date of registration: 27^th^ December 2013); date of enrolment of first patient: 1st August 2013; study retrospectively registered.

## Background

The internet has become one of the main sources of information in many areas of everyday life and has thereby influenced not only the medical field in general, but also the physician-patient interaction [1–3]. Nowadays, many patients research online about health issues and they use the internet to answer specific questions about diseases and therapies, but also to find out rankings and reviews about their hospital and physicians [1, 4]. It has been shown that medical websites can increase patient knowledge, interest in their health status and level of patients’ compliance [5, 6]. In this rapidly growing field of opportunities, even internet-based patient–provider communication services (IPPC), allowing patients to communicate electronically with professionals about medical issues, are a promising but so far lowly enrolled service [7]. For example, oncological patients benefited from internet-based, interactive health communication applications that provide individual support by electronic communication with medical experts [8]. Surgeons try to use online postoperative care planning applications to improve patient access while increasing their efficiency [9].

Patients seek information online in many different medical disciplines. For example, women undergoing repeat caesarean sections frequently used the internet for decision-making if vaginal birth after caesarean section is an available option [10]. Data are also available regarding the preoperative setting before orthopaedic surgery [11], in Crohns disease [12], or oncology [8].

Hence, an increasing number of health institutions, offer more and more information especially geared towards their patients online. The amount of medical information available in the internet seems to be sufficient to satisfy most users and to contribute in reducing confusion about their personal medical issues [13, 14].

However, internet-based health information comes from a wide range of different sources. It may feature highly technical language, as was for example recently shown in the field of regional anaesthesia [15], and the quality, accuracy, and safety of some health information available in the internet can even be suspect [3, 8, 16–18]. Further, there are inconsistent reports about availability and usability of internet based information for special groups of patients. There are data showing age, level of education or income e.g. as limiting factors [19], whereas others report about more general availability [3].

There is little known about the internet-based data gathering behaviour of patients in the preoperative setting. A data collection in patients after cardiac surgery showed that one fifth of the surveyed 80 patients researched online information preoperatively [20]. A study with a very similar structure to ours showed that about 40% of their 877 enrolled patients had utilised the internet preoperatively to obtain medical information [21]. It is unclear to what extent and what specific contents patients research online, especially regarding their upcoming anaesthetic.

We conducted this patient survey to determine to what level and how our patients utilise the internet before an elective procedure under anaesthesia.

Aim of this study was to identify factors associated with patients using the internet to gather information about their upcoming hospital stay and surgery in general, and more specifically about anaesthesia.

## Methods

The study was prospectively conducted at the Department of Anaesthesia and Intensive Care at the Kantonsspital Frauenfeld (TG, Switzerland) with the approval of the local ethics committee (Kantonale Ethikkomission, Kanton Thurgau, Switzerland) and was retrospectively registered with the German Clinical Trials Register (www.drks.de; DRKS00005434).

In our institution, per year we perform about 8800 surgical procedures, 8200 of them with the involvement of the anaesthesia department. Of these 8200 procedures, 75% are elective (roughly 6150). Our preoperative anaesthesia clinic evaluates about 3100 patients annually, most of them being referred from general surgery (including plastic and hand surgery), orthopaedics, urology and ENT surgery. These patients undergo procedures in an ambulatory outpatient setting or as day of surgery admissions. Patients are usually referred to the preoperative clinic by the surgeon after the decision for a surgical procedure involving anaesthesia has been made. In the meantime, some of the patients see their primary practitioner in preparation, e.g. to draw blood samples. Patients are then seen by the anaesthesiologist in the preoperative clinic, roughly 2–10 days before the scheduled surgical procedure.

Patient selection criteria for this study were age > 18 years, elective procedure with anaesthesia involvement, anaesthesia informed consent performed in anaesthesia preoperative clinic, and sufficient knowledge of the German language. Patients were not eligible prior to an emergency procedure or when they refused to take part in the survey. One thousand consecutive patients that met the criteria were asked if they would participate in this voluntary survey. By completing the study questionnaire, patients expressed their agreement for participation. The further participation in the survey happened anonymously. The sample size was determined to include approximately as much patients as in a similar study in the United States [21], speculating about a rather high response rate in our setting.

The questionnaire was based on the one that was used in the mentioned study [21]. It was translated to German and adjusted to the conditions in Switzerland with the permission of the publishing journal and the corresponding author. The questionnaire included 20 questions that targeted characteristics of the patients’ age, and education. As the Swiss educational system is rather complex, higher education was defined as high school graduation (Matura) or higher. The questions also included their general computer and internet habits, and their specific internet usage to obtaining information relevant for their upcoming procedure (hospital, surgeon, procedure, disease), and the associated anaesthetic. Specifically, the patients having used the internet to gather information about their anaesthetic were asked if they looked up, what an anaesthetist is, what kinds of anaesthesia were possible for them, did they need tracheal intubation, what about food intake and medication use prior to the procedure.. Patients having used the internet to obtain information were asked which internet sites they used and if they felt they did get the wanted information. Patients not having done so were asked for the reason.

Primary outcome were patients using the internet to gather any medical information related to their upcoming hospital stay, secondary outcome the patients using the internet to gather information regarding the upcoming anaesthesia.

### Statistics

Patient characteristics are presented as number (n) and percentage (%). Univariate and multivariate logistic regression was used to identify factors associated with internet use. Results are presented in odds ratios (OR) and 95% confidence intervals (95% CI).

All statistical analyses were performed using STATA 13 (StataCorp. 2013. *Stata Statistical Software: Release 13*. College Station, TX: StataCorp LP).

## Results

Out of the 1000 patients asked, 82% (*n* = 815) agreed to participate in the study. Ninety-seven percent of these 815 patients were classified as American Society of Anesthesiology (ASA) physical status of 1 or 2 (ASA 1 = normal, healthy patient; ASA 5 = moribund patient, expected to dye without operation) and 99% underwent a surgical procedure with a minor or moderate risk level. Approximately one third of the procedures were planned to take place in an outpatient setting, the remaining as day of surgery admissions. Eighty-three percent (*n* = 676) of the patients had already had experience with previous anaesthetics (Table 1).

**Table 1:** Patient demographics, overall internet habits and topics searched for by participating patients (*n* = 815)

Patient demographics and internet habits			
Parameter		*n*	%
Age (years)	18–44	355	44
	45–60	250	31
	61–80	204	25
	>80	6	1
Sex	male	441	54
	female	374	46
ASA physical status	1	395	48
	2	396	49
	3	24	3
	4	0	0
	5	0	0
Level of education	apprenticeship etc.	610	75
	higher education ^a^	205	25
Previous anaesthetic	yes	676	83
	no	139	17
Average internet usage per week (h)	never	115	14
	< 5	300	37
	5–10	215	26
	10–15	83	10
	> 15	102	13
Postoperatively planned as inpatient	yes	551	68
	no	264	32
Type of surgery	minor	499	61
	moderate	306	38
	major	10	1
Topics researched online preoperatively			
	Surgery	209	26
	Surgeon	51	6
	Hospital	94	12
	Disease	257	32
	Anaesthetic	55	7

*ASA* American Society of Anesthesiology (physical status 1 = normal, healthy patient; physical status 5 = moribund patient, expected to dye without operation). ^a^ Higher education defined as high school graduation (Matura) or higher

Eighty-six percent (*n* = 700) of the people indicated that they regularly use the internet (Table 1), 14% (*n* = 115) did not use the Internet at all or have access. The average age of those internet users was 46 years (Table 1), 25% (*n* = 205) of all respondents and 28% (*n* = 199) of the internet users completed some form of higher education. About a third of the patients (*n* = 279) used the internet to obtain information about their medical condition, and 26% used it to gain insight into their upcoming procedure. Specific information about the hospital or surgeon was less frequently sought with 12, and 6% respectively (Table 1).

In univariate analyses, status of higher education and more extensive usual internet using habit were associated with internet use. In contrast, higher age, ASA physical status 2, and experience from previous anaesthetics were associated with not using the internet for that purpose (Table 2). After adjusting for all other covariates, more extensive usual internet using habit, and planned moderate in contrast to minor surgery were independently associated with internet use. Experience from previous anaesthetics was associated with not doing so (Table 2). Statistical analysis of the patients having used the internet to gather information about their upcoming anaesthetic did not reveal any significant differences compared to the patients having used the internet to gather more general information (Table 3).

**Table 2:** Factors associated with patients using the internet to gather information about their upcoming surgery in general. Patients having used the internet to do so (any internet) vs. patients not having done so (no internet). Data are percentage (number) and 95% confidence interval for univariate analysis (crude OR) and multivariate regression analysis (adjusted OR)

		internet use preoperatively			
		any	no		
		% (n)	% (n)	crude OR (95% CI)	adjusted OR (95% CI)
total *n* = 815		41% (334)	59% (481)		
Age (years)	18–44	49.4% (177)	50.6% (181)	reference	reference
	45–60	41.2% (103)	58.8% (147)	0.72 (0.52–0.99)	0.98 (0.67–1.41)
	61–80	26.9% (54)	73.1% (147)	0.37 (0.26–0.55)	0.75 (0.47–1.18)
	> 80	0% (0)	100% (6)	n/a	n/a
Gender	male	40.4% (178)	59.6% (263)	reference	reference
	female	41.7% (156)	58.3% (218)	1.06 (0.80–1.40)	1.24 (0.91–1.69)
ASA physical status	1	45.1% (178)	54.9% (217)	reference	reference
	2	37.9% (150)	62.1% (246)	0.74 (0.56–0.99)	1.01 (0.72–1.42)
	3	25.0% (6)	75.0% (18)	0.4 (0.16–1.05)	0.69 (0.22–2.18)
Level of education	apprenticeship etc.	37.4% (228)	62.6% (382)	reference	reference
	higher education ^a^	51.7% (106)	48.3% (99)	1.78 (1.30–2.47)	1.34 (0.95–1.89)
Previous anaesthetic	no	56.8% (79)	43.2% (60)	reference	reference
	yes	37.7% (255)	62.3% (421)	0.46 (0.32–0.67)	0.56 (0.38–0.84)
Average internet usage per week (h)	0	7.0% (8)	93.0% (107)	reference	reference
	0 to 5	38.0% (114)	62.0% (186)	8.20 (3.85–17.45)	7.13 (3.28–15.52)
	5 to 10	47.9% (103)	52.1% (112)	12.3 (5.71–26.48)	10.24 (4.61–22.76)
	10 to 15	59.0% (49)	40.1% (34)	19.27 (8.31–44.70)	15.59 (6.41–37.9)
	more than 15	58.8% (60)	41.2% (42)	19.11 (8.42–43.36)	14.34 (5.96–34.50)
Postoperatively planned as outpatient	no	41.7% (110)	58.3% (154)	reference	reference
	yes	40.1% (224)	59.4% (327)	0.96 (0.71–1.29)	0.82 (0.58–1.16)
Type of surgery	minor	61.0% (304)	39.1% (195)	reference	reference
	moderate	55.9% (171)	44.1% (135)	1.23 (0.92–1.64)	1.70 (1.20–2.43)
	major	60.0% (6)	40.0% (4)	1.04 (0.29–3.73)	2.75 (0.61–12.35)

*OR* odds ratio, *ASA* American Society of Anesthesiology (physical status 1 = normal, healthy patient; physical status 5 = moribund patient, expected to dye without operation). ^a^ Higher education defined as high school graduation (Matura) or higher. *n/a* not applicable

**Table 3:** Factors associated with patients using the internet to gather information about their upcoming anaesthetic. Patients having used the internet to do so (anaesthetic) vs. patients having used the internet to obtain information for other questions (other). Data are percentage (number) and 95% confidence interval for univariate analysis (crude OR) and multivariate regression analysis (adjusted OR)

		internet use preoperatively			
		anaesthetic	other		
		% (n)	% (n)	crude OR (95% CI)	adjusted OR (95% CI)
total *n* = 334		16.5% (55)	83.5% (279)		
Age (years)	18–44	17.5% (31)	82.5% (146)	reference	reference
	45–60	14.6% (15)	85.4% (88)	0.80 (0.41–1.57)	1.22 (0.58–2.58)
	61–80	16.7% (9)	83.3% (45)	0.94 (0.42–2.16)	1.50 (0.56–4.03)
	> 80	0% (0)	0% (0)	n/a	n/a
Gender	male	16.8% (30)	83.2% (148)	reference	reference
	female	16.0% (25)	84.0% (131)	0.94 (0.53–1.68)	1.17 (0.62–2.19)
ASA physical status	1	18.5% (33)	81.5% (145)	reference	reference
	2	13.3% (20)	86.7% (130)	0.68 (0.37–1.24)	0.56 (2.78–1.15)
	3	33.3% (2)	66.7% (4)	2.20 (0.39–12.50)	3.75 (0.50–28.04)
Level of education	apprenticeship etc.	16.7% (38)	83.3% (190)	reference	reference
	higher education ^a^	16.0% (17)	84.0% (89)	0.96 (0.51–1.78)	0.81 (0.42–1.58)
Previous anaesthetic	no	20.2% (15)	79.8% (63)	reference	reference
	yes	15.3% (39)	84.7% (216)	0.71 (0.37–1.36)	0.81 (0.4–1.65)
Average internet usage per week (h)	0	25.0% (2)	75.0% (6)	reference	reference
	0 to 5	7.9% (9)	92.1 (105)	0.26 (0.05–1.46)	0.20 (0.33–1.26)
	5 to 10	14.6% (15)	85.4% (88)	0.51 (0.09–2.78)	0.44 (0.08–2.54)
	10 to 15	22.5% (11)	77.6% (38)	0.87 (0.15–4.92)	0.82 (0.14–5.0)
	more than 15	30.0% (18)	70.0% (42)	1.29 (0.24–6.99)	1.22 (0.20–7.28)
Postoperatively planned as outpatient	no	18.2% (20)	81.8% (90)	reference	reference
	yes	15.6% (35)	84.4% (189)	0.83 (0.46–1.52)	0.87 (0.42–1.78)
Type of surgery	minor	15.9% (31)	84.1% (164)	reference	reference
	moderate	17.8% (24)	82.2% (111)	1.15 (0.64–2.5)	1.36 (0.66–2.80)
	major	100% (4)	0% (0)	n/a	n/a

*OR* odds ratio, *ASA* American Society of Anesthesiology (physical status 1 = normal, healthy patient; physical status 5 = moribund patient, expected to dye without operation). ^a^ Higher education defined as high school graduation (Matura) or higher. *n/a* not applicable

The vast majority of the patients that participated (93%, *n* = 760) did not utilise the internet to obtain information about their upcoming anaesthetic. Asked for the reasons of doing so, 84% (*n* = 636) of those patients reported that they had no further unanswered questions after the informed consent discussion with the surgeon. 7% (*n* = 50) felt that they had no further need for information because of previous anaesthetics. For the remaining patients, an internet search was either not possible because they did not readily have internet access (1%, *n* = 11) or because they did not know on which websites the information could have been obtained (4%, *n* = 33).

Of those patients who used the internet for anaesthesia relevant information, 76% (*n* = 41), utilised a search engine, 28% (*n* = 15) visited the website of our hospital and 26% (*n* = 14) the website of a relevant professional organization (e.g. the Swiss Society of Anaesthesiology and Reanimation). Eighty-seven percent (*n* = 47) of the patients felt that they were able to find sufficient answers for their questions regarding the upcoming anaesthetic. Questions patients tried to answer regarding the anaesthetic were about possible kind of anaesthetic (85%, 46 patients), food intake being allowed (48%, *n* = 26), the need for tracheal intubation (46%, *n* = 25), and how to deal with their medication (37%, *n* = 20). 12% (*n* = 22) searched for general information about what is an anaesthetist.

Thirty-four percent (*n* = 262) of the participants not using the internet specifically in advance of their upcoming procedure would have visited a web site, if their medical team had informed them about where to find relevant content.

## Discussion

The number of so-called “e-patients”, patients who routinely use the internet to gather information about their health care is steadily increasing [2]. Patients consult the internet to answer their questions about health concerns either in addition or instead of the conventional interaction with their physician. Little data on internet use exists for the preoperative setting, especially with regards to the search for information concerning the anaesthetic portion [21].

In this study patients were interviewed using a questionnaire as part of their anaesthesia preoperative clinic visit before an elective surgical procedure. The emphasis was both on the general internet usage habits and the more specific search behaviour with regard to the upcoming procedure and the anaesthetic.

The participation rate in this voluntary survey was fairly high at over 80%. The main finding of the survey was that only a few patients had used the internet in order to obtain general perioperative relevant information, and even fewer had used it to become more informed about the anaesthetic portion. However, many patients stated that they would have appreciated and made use of information towards trustworthy and valuable internet sites.

Patients do use the internet in the perioperative setting. A data collection in patients after cardiac surgery showed that one fifth of the surveyed 80 patients researched online information preoperatively [20]. A study with a very similar structure to ours showed that about 40% of their 877 enrolled patients had utilised the internet preoperatively to obtain medical information [21]. It appears that the anaesthesia portion plays an even less important role as far as the need for information in the perioperative setting goes. In the above cited study by Kurup et al. [21] there were just 4% of patients who were interested in getting information on this topic from the internet. Our study resulted in fairly similar results, with just under a third of the patients stating that they used the internet to obtain information relevant to their disease and the pending procedure, and only less than 10% doing so to get more informed about their upcoming anaesthetic. These figures do seem fairly low, especially when compared to customers’ usage of the internet to research information pertinent to decisions on the topics of travel or telephone; where data from Germany show a 70% overall rate (https://www.bitkom.org/Presse/Presseinformation/Pressemitteilung_4300.html).

We surveyed our patients for the reasons why they had not used the internet to search for pertinent information. Statistically, the only factor remaining relevant in the multiple regression analysis for not having used the internet was having experience from a previous anaesthetic, which most probably includes experience with surgery and hospital set-up in general. Asking the patients more explicitly, revealed the fact that they already felt well informed about the anaesthetic after the informed consent discussion with the surgeon, even though anaesthesia pre-information is not an essential part of this. Similarly, in our system, many patients see their primary care physician for additional preoperative testing prior to their preoperative clinic visit. This physician contact could also contribute to anaesthesia relevant information.

Another important reason for the low rate of internet usage could be associated to age-related limitations, even if in this study age not proved to be an independent factor. However, 14% (*n* = 115) of the patients did not use the internet at all or did not have access to it. This subgroup of patients was on average 62 years old and only 5% of those (*n* = 6) reported to have had a higher education. In contrast, the average age of internet users in our patient population was lower (46 years) and the rate of higher education was higher (28%; 199 patients). The so-called “e-patient” seems to be of younger age and better education. Hence, a solely internet-based patient information strategy for the preoperative period could miss important subgroups and should be viewed critically [19, 22].

Of the patients who utilised the internet to inform themselves about their upcoming anaesthesia, 85% were content with what they found and felt sufficiently informed. The majority of them used conventional search engines. It is very likely that the nature and quality of the information found varied greatly [16, 17, 23]. It remains unclear how involved the government, health organizations and hospitals ideally should be in order to improve patient information and satisfaction. A third of the patients, who did obtain information via the internet, stated that they gathered it from the website of our hospital, and professional organizations. The Swiss Society of Anesthesiology and Reanimation (SGAR) provides such a professionally managed patient information site (www.anaesthesie-info.ch). A significant proportion of our patients (34%), who had not made use of the internet, were very open to according suggestions from medical professionals. The anaesthesiologist that sees the patient in the preoperative clinic days before the actual anaesthetic and procedure happens could provide patients with credible internet resources in a standardised fashion. A card of information directing patients to trustworthy internet sites might be a good strategy for doing so.

Despite using a very similar questionnaire, the comparison of our results with the ones of Kurup et al. [21] produces some interesting differences. This can be partly due to different health care environments and culture (i.e. trust in surgeon and system, ease of access to a primary care physician, education level), and habits of internet use (importance of rankings, etc.). Additionally, it is also not entirely clear how comparable the two patient groups are. This is reflected for one in the response rate that at over 80% was significantly higher in our study than the 30% reported by Kurup et al. The authors did not further comment the possible influence of a low response rate on their results. For sure, there are culture and society aspects influencing also this part of health care, so comparisons between different settings have to been interpreted carefully.

Another reason for the relatively low portion of patients that conducted a more thorough pre-operative internet search could also be attributed to the fact that the patients who participated in our study were generally in good health and had to undergo relatively minor procedures. This is reflected in the fact that patients planned to undergo moderate in comparison to minor surgery were more likely to inform themselves in the internet.

There are of course limitations to our study. Higher education was defined very crudely because of the complexity of the Swiss educational system. The questions about what exactly patients did look for regarding the anaesthetic did not check for fear or uneasiness and could be held more open. Also, the time frame of our questions was not very clear. So it is not really known when a respondent had looked up information (e.g. within the last 12 months, etc. or in the 4 weeks prior to the procedure). Another interesting question not answered so far would be to find out if there might be a difference in perioperative dealing with patients between those who used the internet and those who did not. Such a difference could warrant spreading the culture of searching trustworthy sites in order to improve the medical service.

## Conclusions

The majority of our study group did not use the internet to obtain additional information about their anaesthetic before the upcoming elective procedure. Based on our results, exclusively web-based patient information systems on anaesthesia would not be successful. However, patients are open to and would appreciate suggestions regarding relevant online resources by a healthcare provider.

## Abbreviation

ENT: Ear nose and throat surgery
